# Supplementary figures and images for: Time-varying associations between an exposure history and a subsequent health outcome: a landmark approach to identify critical windows
Source: BMC Med Res Methodol. 2021 Nov 27;21:266. doi: 10.1186/s12874-021-01403-w (PMC8627635; doi:10.1186/s12874-021-01403-w)

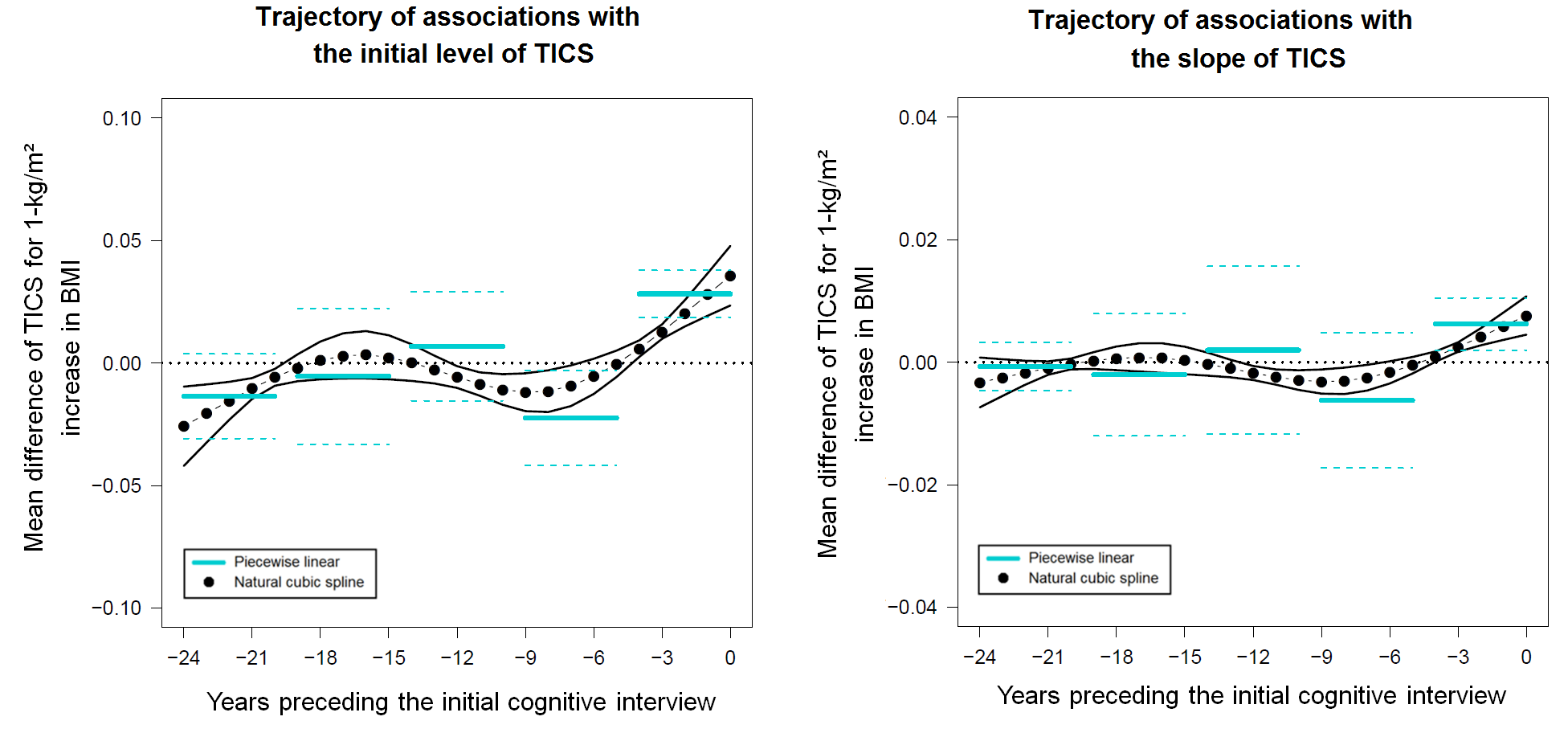

Supplement: Supplementary file 1 — Additional file 1 eFigure 1. Trajectories of associations between the body mass index history in the 24 years prior to the first cognitive interview on the initial level (left panel) or the slope (right panel) of the Telephone Interview for Cognitive Status (TICS) score approximated by natural cubic splines (in black) or 5-year piecewise constants (in blue) in the Nurses’ Health Study (N=19,381), United States (1976-2000). 95% confidence intervals were obtained by parametric bootstrap with 500 replicates. [file 12874_2021_1403_MOESM1_ESM.png]

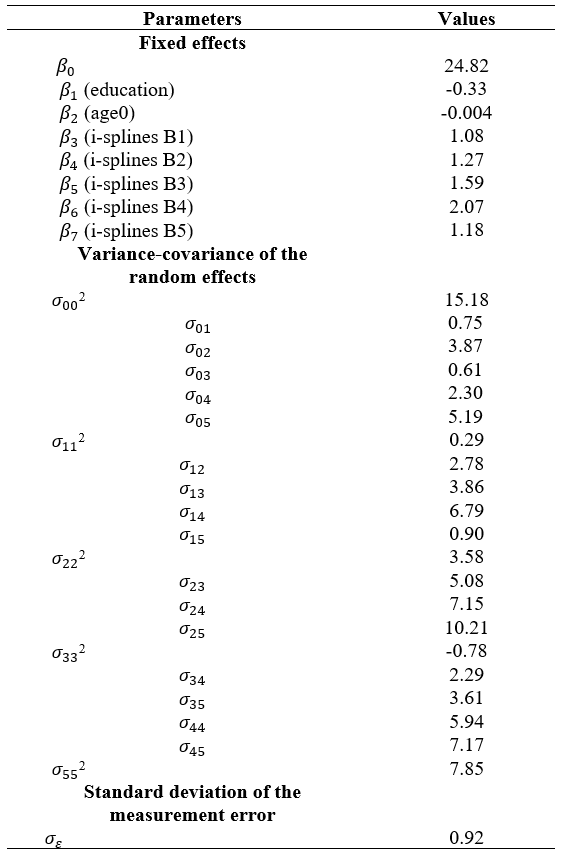

Supplement: Supplementary file 2 — Additional file 2 eTable 1. Parameter values used for the generation of the exposure data in the main simulation scenario. [file 12874_2021_1403_MOESM2_ESM.png]

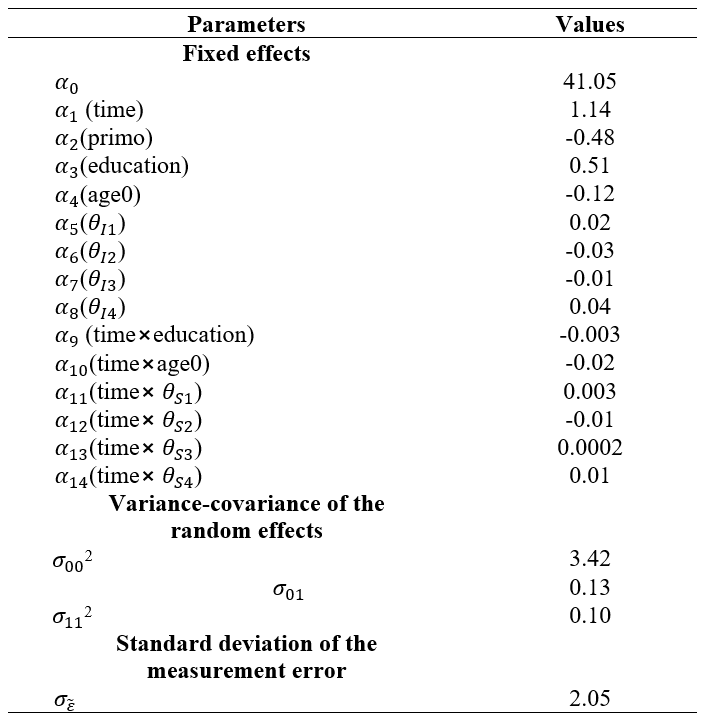

Supplement: Supplementary file 3 — Additional file 3 eTable 2. Parameter values used for the generation of the outcome data in the main simulation scenario. [file 12874_2021_1403_MOESM3_ESM.png]

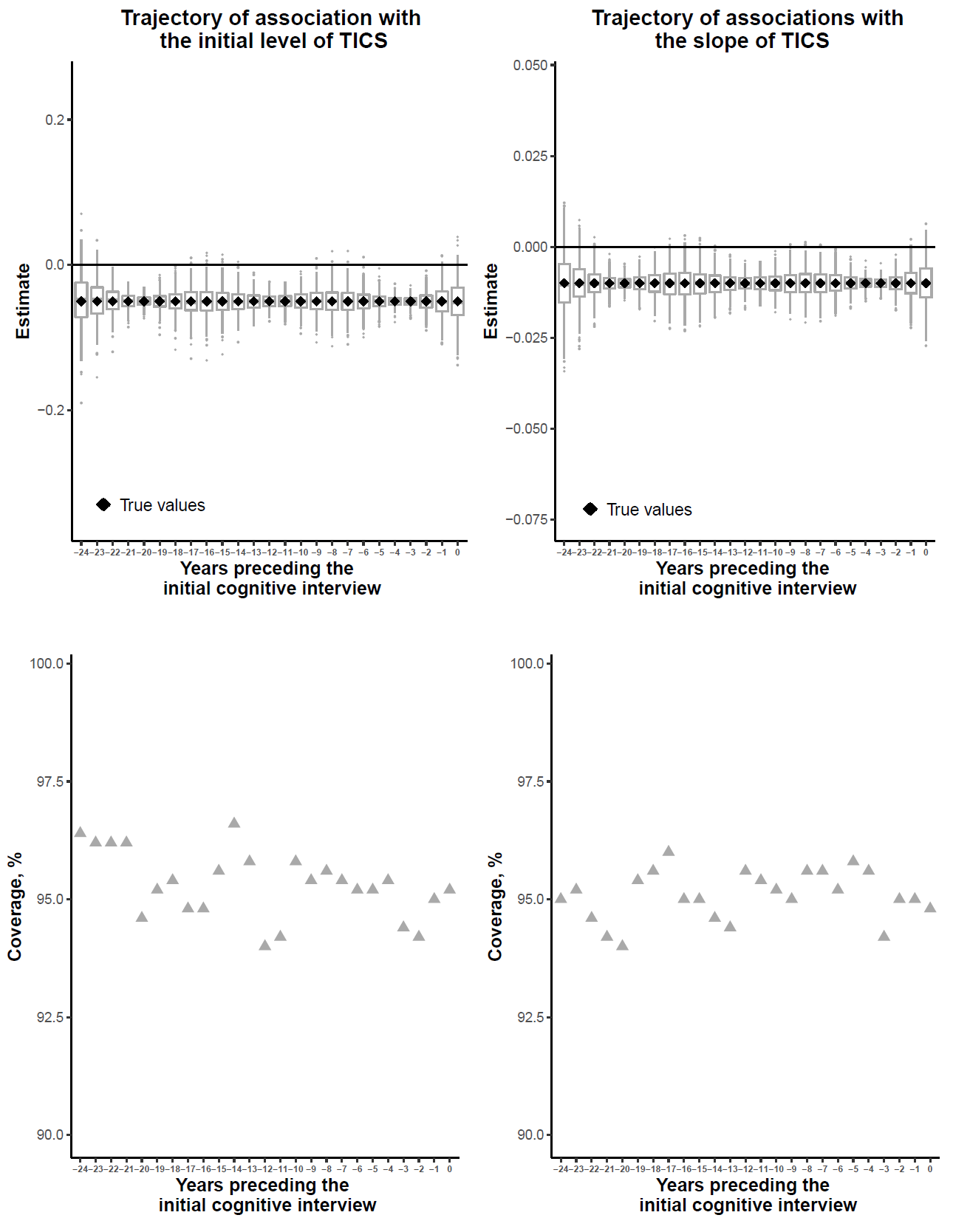

Supplement: Supplementary file 4 — Additional file 4 eFigure 2. Boxplots of the trajectory of association between the exposure history over the 24 years prior to the initial health outcome assessment on the initial level (top left panel) and slope (top right panel) of the outcome of interest across 500 simulations of 1000 subjects each, and corresponding coverage rates (low panels) for Scenario A (constant effect). [file 12874_2021_1403_MOESM4_ESM.png]

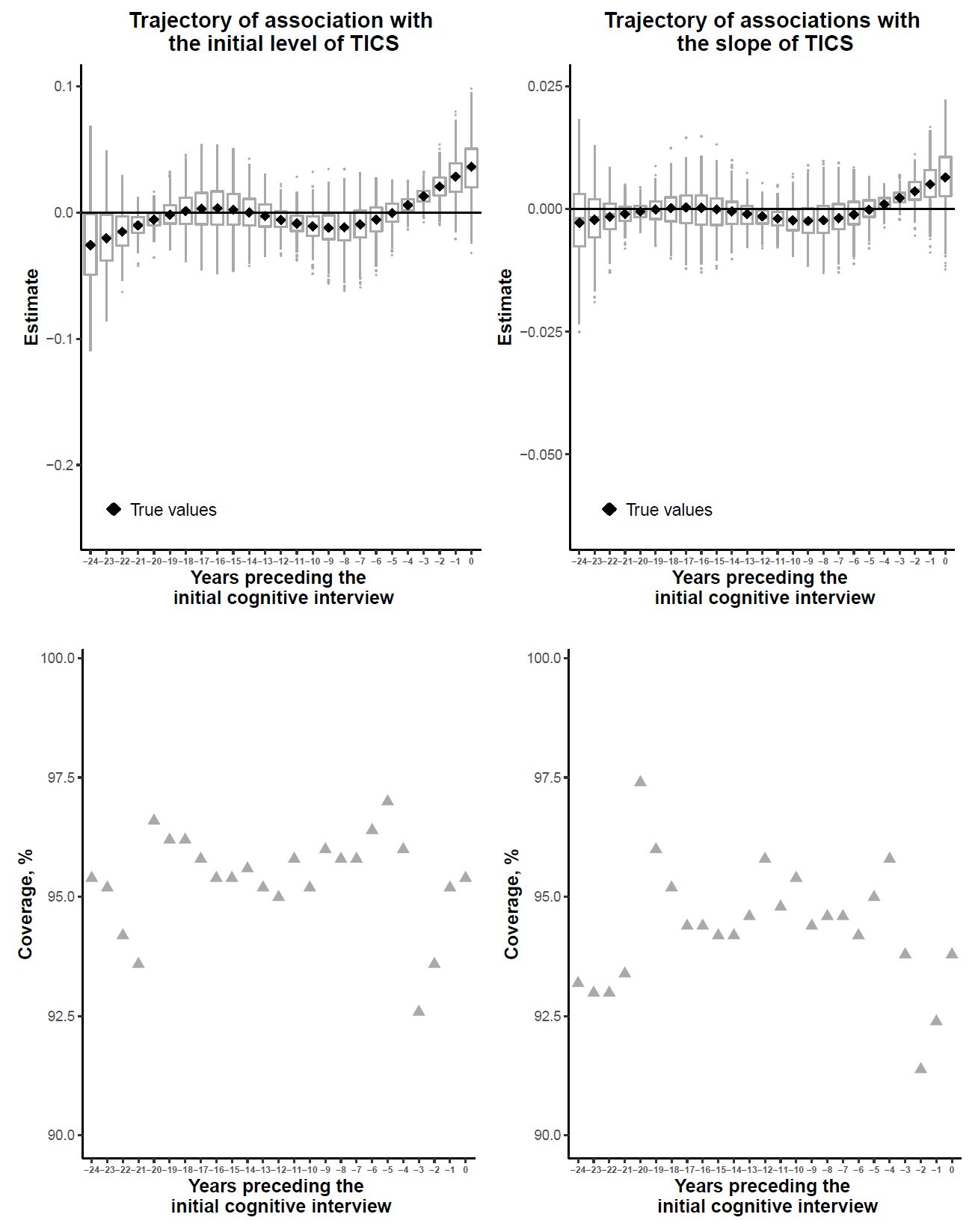

Supplement: Supplementary file 5 — Additional file 5 eFigure 3. Boxplots of the trajectory of association between the exposure history over the 24 years prior to the initial health outcome assessment on the initial level (top left panel) and slope (top right panel) of the outcome of interest across 500 simulations of 1000 subjects each, and corresponding coverage rates (low panels) for Scenario C (effect mimicking the associations between BMI and TICS in the Nurses’ Health Study). [file 12874_2021_1403_MOESM5_ESM.png]

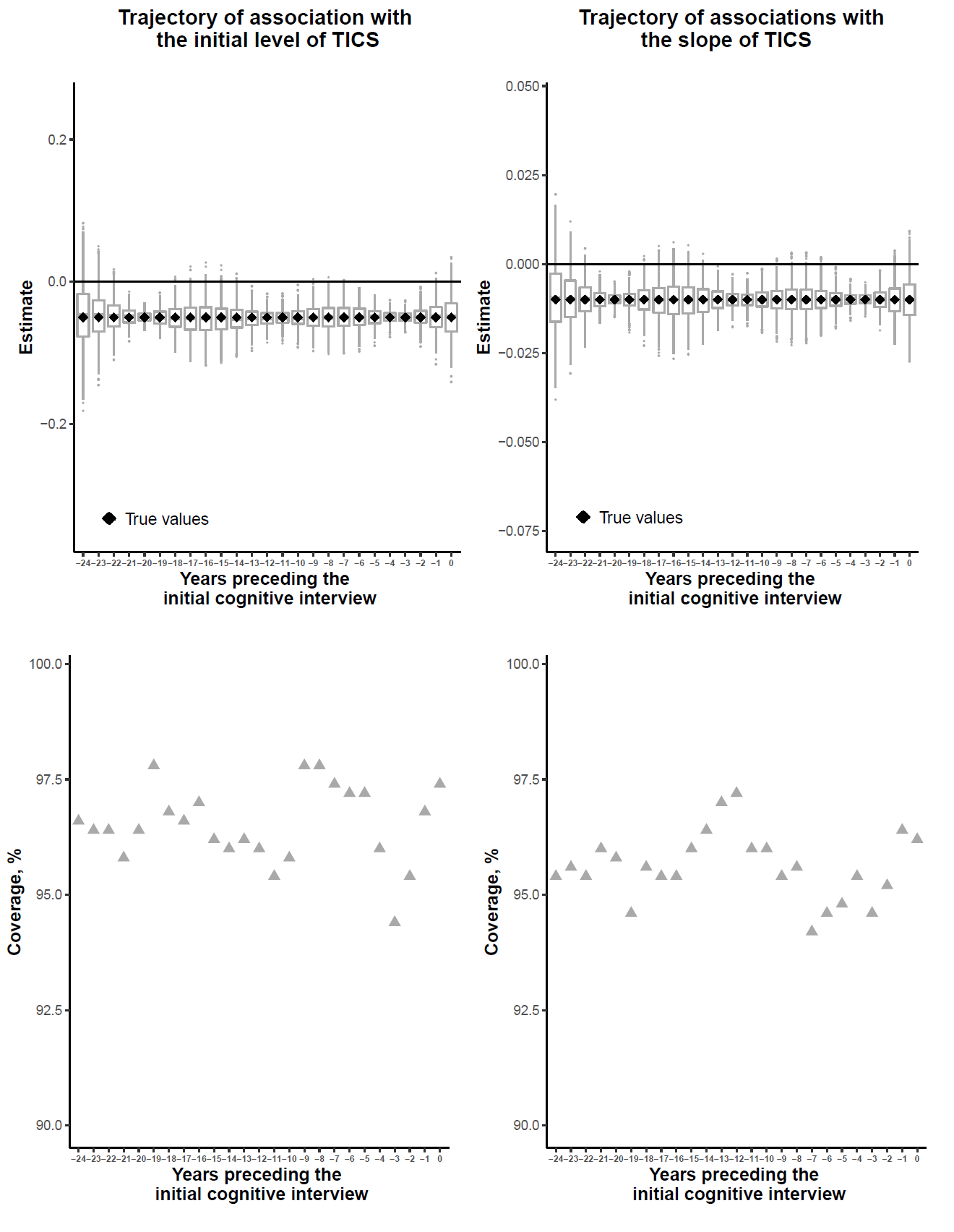

Supplement: Supplementary file 6 — Additional file 6 eFigure 4. Boxplots of the trajectory of association between the exposure history over the 24 years prior to the initial health outcome assessment on the initial level (top left panel) and slope (top right panel) of the outcome of interest when considering less repeated information for exposure (i.e., measured every 4 years instead of every 2 years) across 500 simulations of 1000 subjects each, and corresponding coverage rates (low panels) for Scenario A (constant effect). [file 12874_2021_1403_MOESM6_ESM.png]

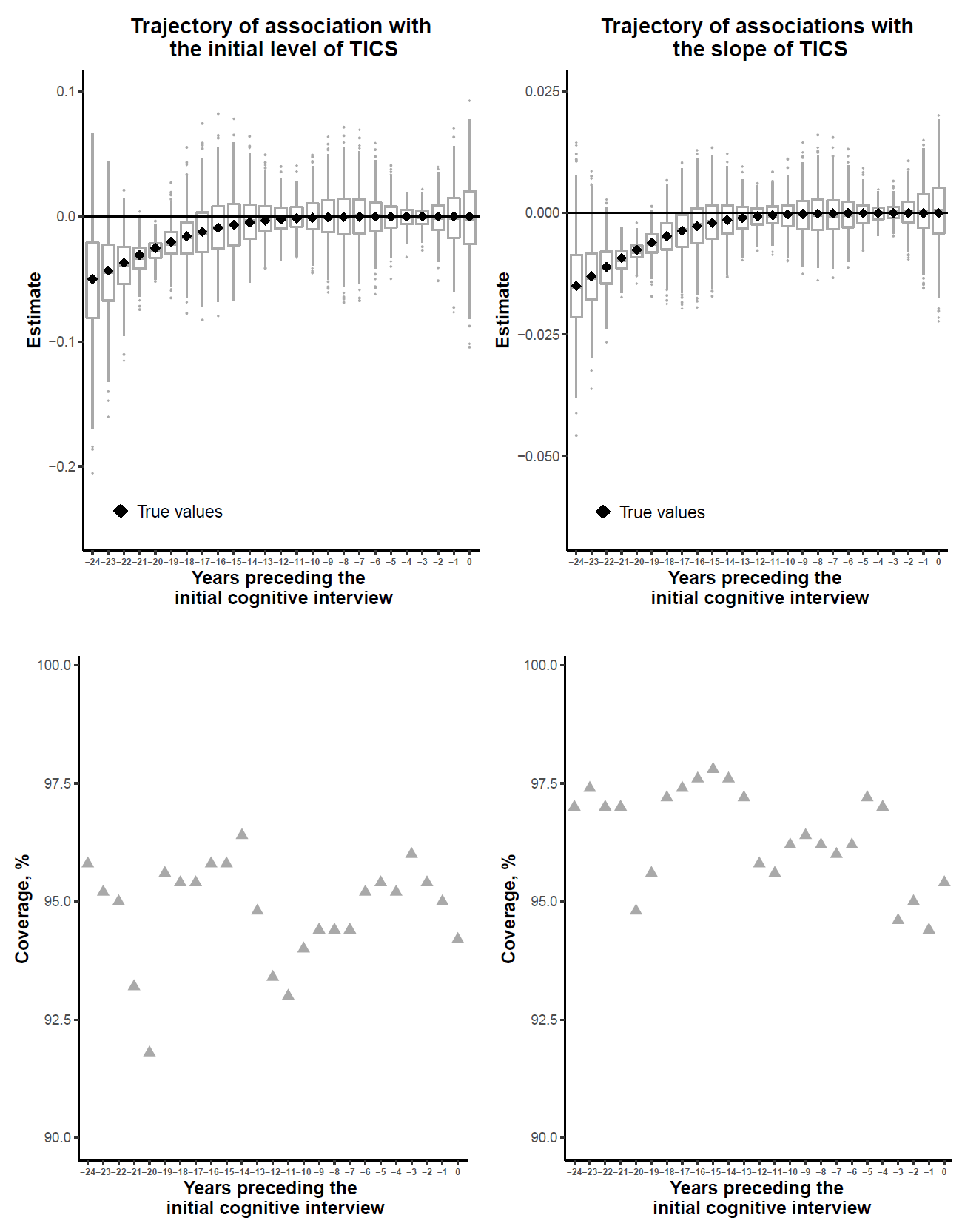

Supplement: Supplementary file 7 — Additional file 7 eFigure 5. Boxplots of the trajectory of association between the exposure history over the 24 years prior to the initial health outcome assessment on the initial level (top left panel) and slope (top right panel) of the outcome of interest when considering less repeated information for exposure (i.e., measured every 4 years instead of every 2 years) across 500 simulations of 1000 subjects each, and corresponding coverage rates (low panels) for Scenario B (distant negative effect). [file 12874_2021_1403_MOESM7_ESM.png]

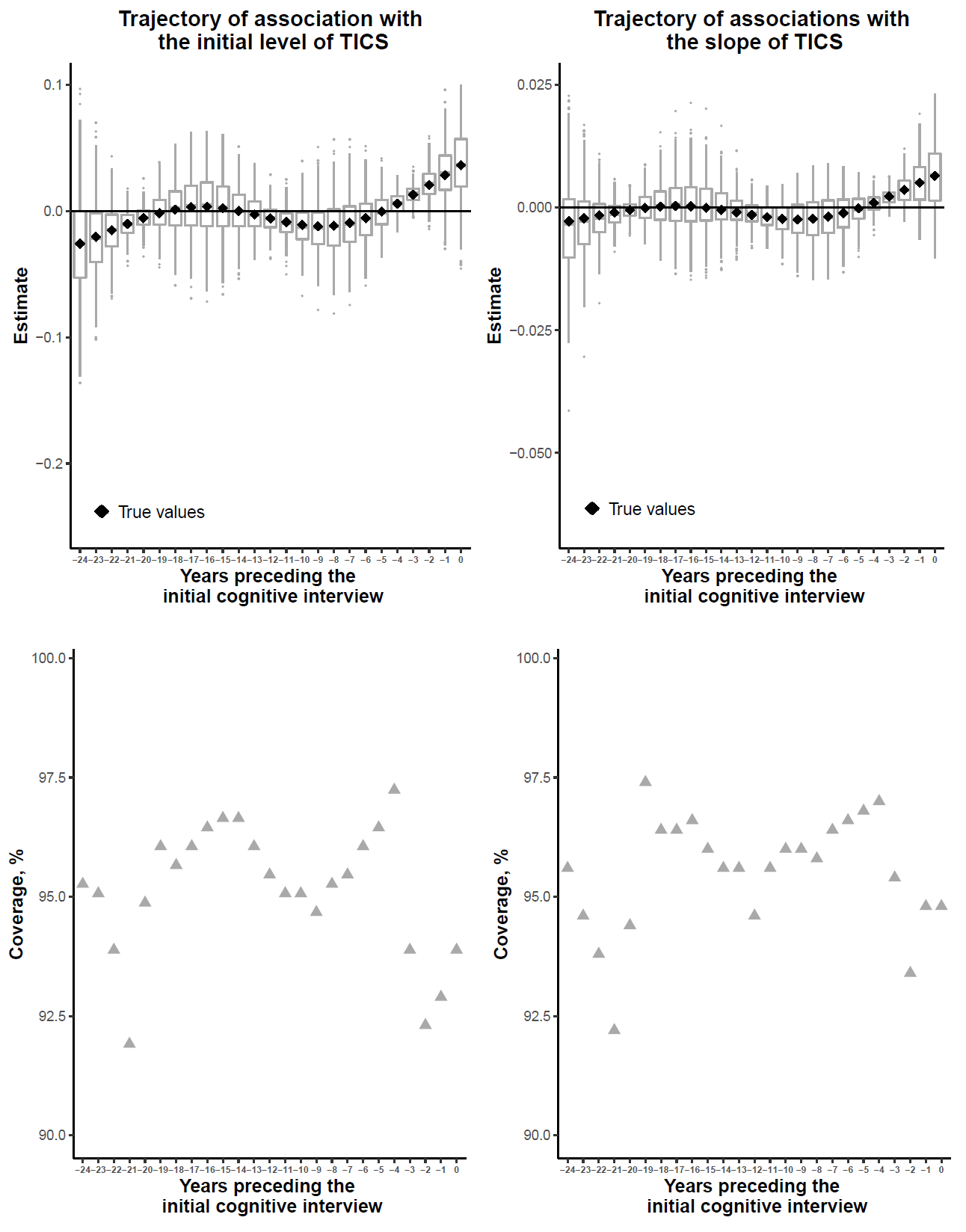

Supplement: Supplementary file 8 — Additional file 8 eFigure 6. Boxplots of the trajectory of association between the exposure history over the 24 years prior to the initial health outcome assessment on the initial level (top left panel) and slope (top right panel) of the outcome of interest when considering less repeated information for exposure (i.e., measured every 4 years instead of every 2 years) across 500 simulations of 1,000 subjects each, and corresponding coverage rates (low panels) for Scenario C (effect mimicking the associations between BMI and TICS in the Nurses’ Health Study). [file 12874_2021_1403_MOESM8_ESM.png]

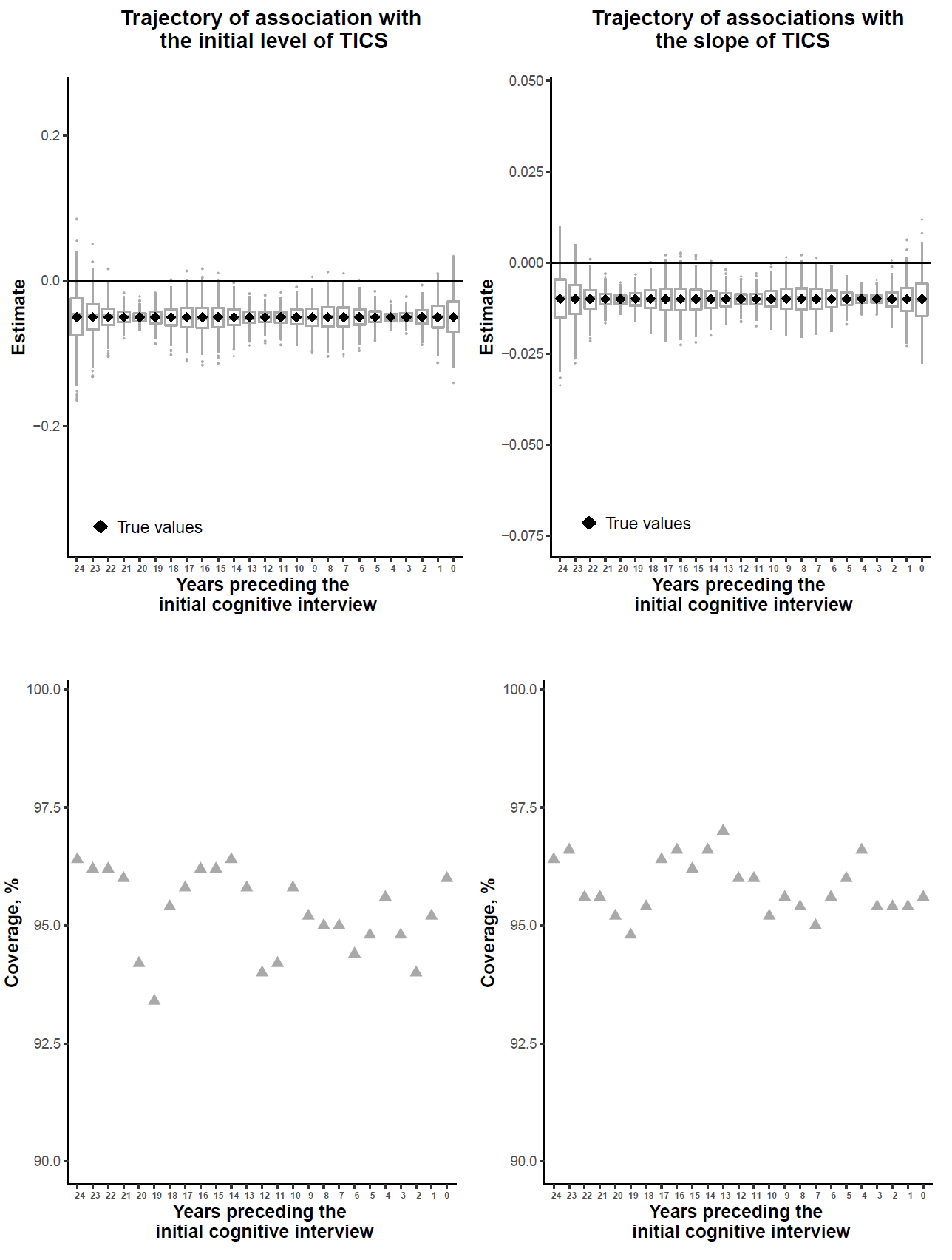

Supplement: Supplementary file 9 — Additional file 9 eFigure 7. Boxplots of the trajectory of association between the exposure history over the 24 years prior to the initial health outcome assessment on the initial level (top left panel) and slope (top right panel) of the outcome of interest when considering a larger proportion of missing data for the exposure (i.e., 20% instead of 10%) across 500 simulations of 1000 subjects each, and corresponding coverage rates (low panels) for Scenario A (constant effect). [file 12874_2021_1403_MOESM9_ESM.png]

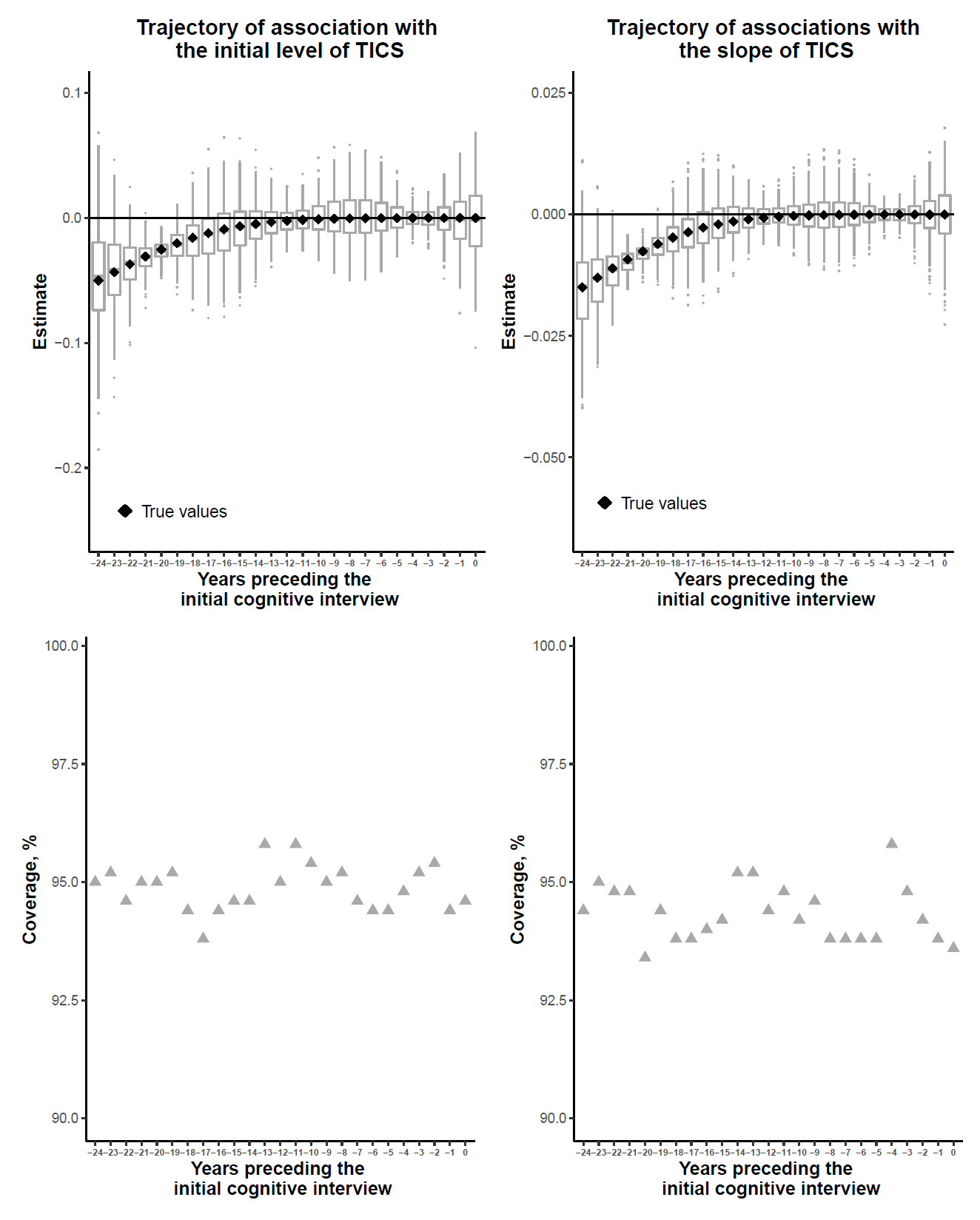

Supplement: Supplementary file 10 — Additional file 10 eFigure 8. Boxplots of the trajectory of association between the exposure history over the 24 years prior to the initial health outcome assessment on the initial level (top left panel) and slope (top right panel) of the outcome of interest when considering a larger proportion of missing data for the exposure (i.e., 20% instead of 10%) across 500 simulations of 1000 subjects each, and corresponding coverage rates (low panels) for Scenario B (distant negative effect). [file 12874_2021_1403_MOESM10_ESM.png]

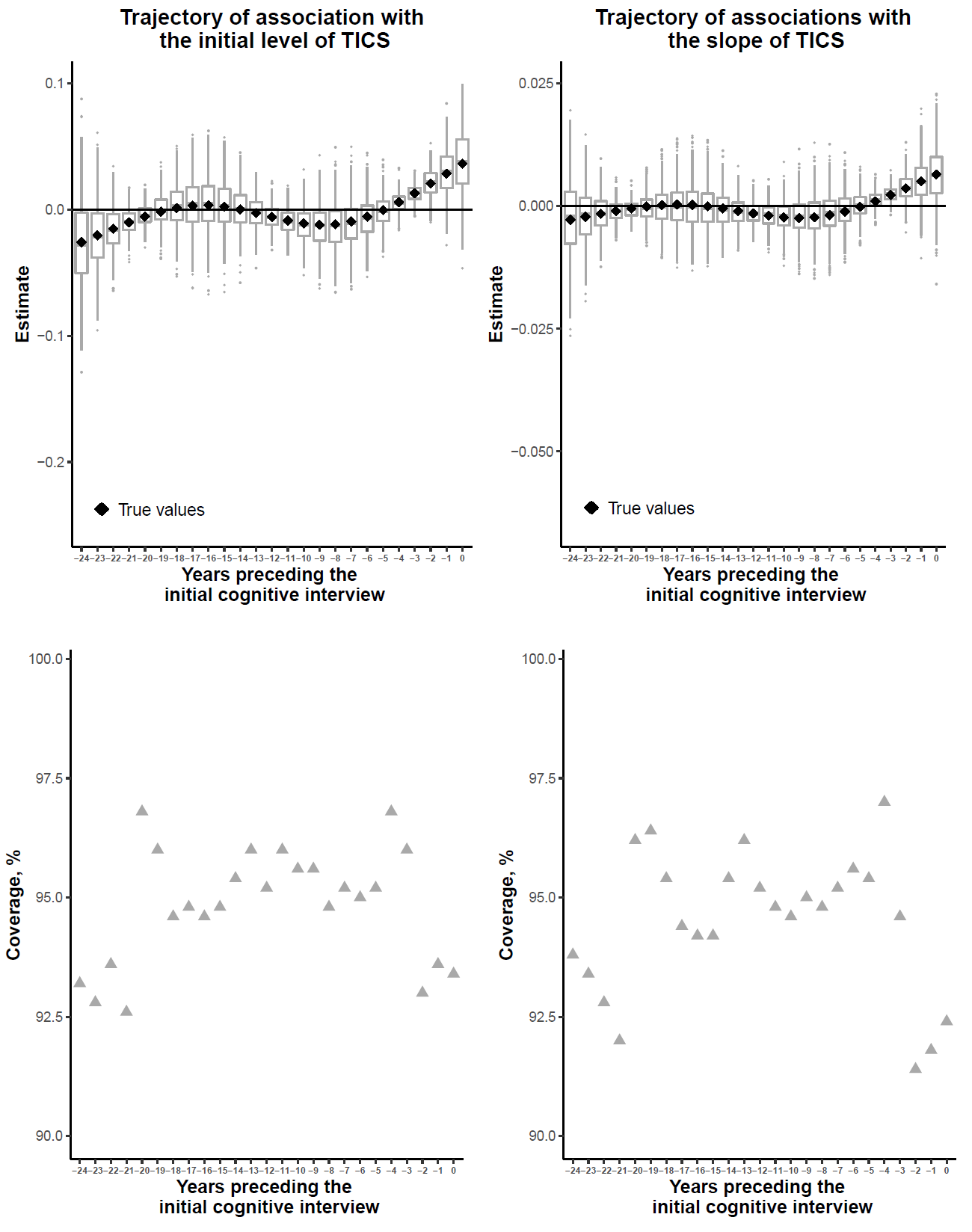

Supplement: Supplementary file 11 — Additional file 11 eFigure 9. Boxplots of the trajectory of association between the exposure history over the 24 years prior to the initial health outcome assessment on the initial level (top left panel) and slope (top right panel) of the outcome of interest when considering a larger proportion of missing data for the exposure (i.e., 20% instead of 10%) across 500 simulations of 1000 subjects each, and corresponding coverage rates (low panels) for Scenario C (effect mimicking the associations between BMI and TICS in the Nurses’ Health Study). [file 12874_2021_1403_MOESM11_ESM.png]

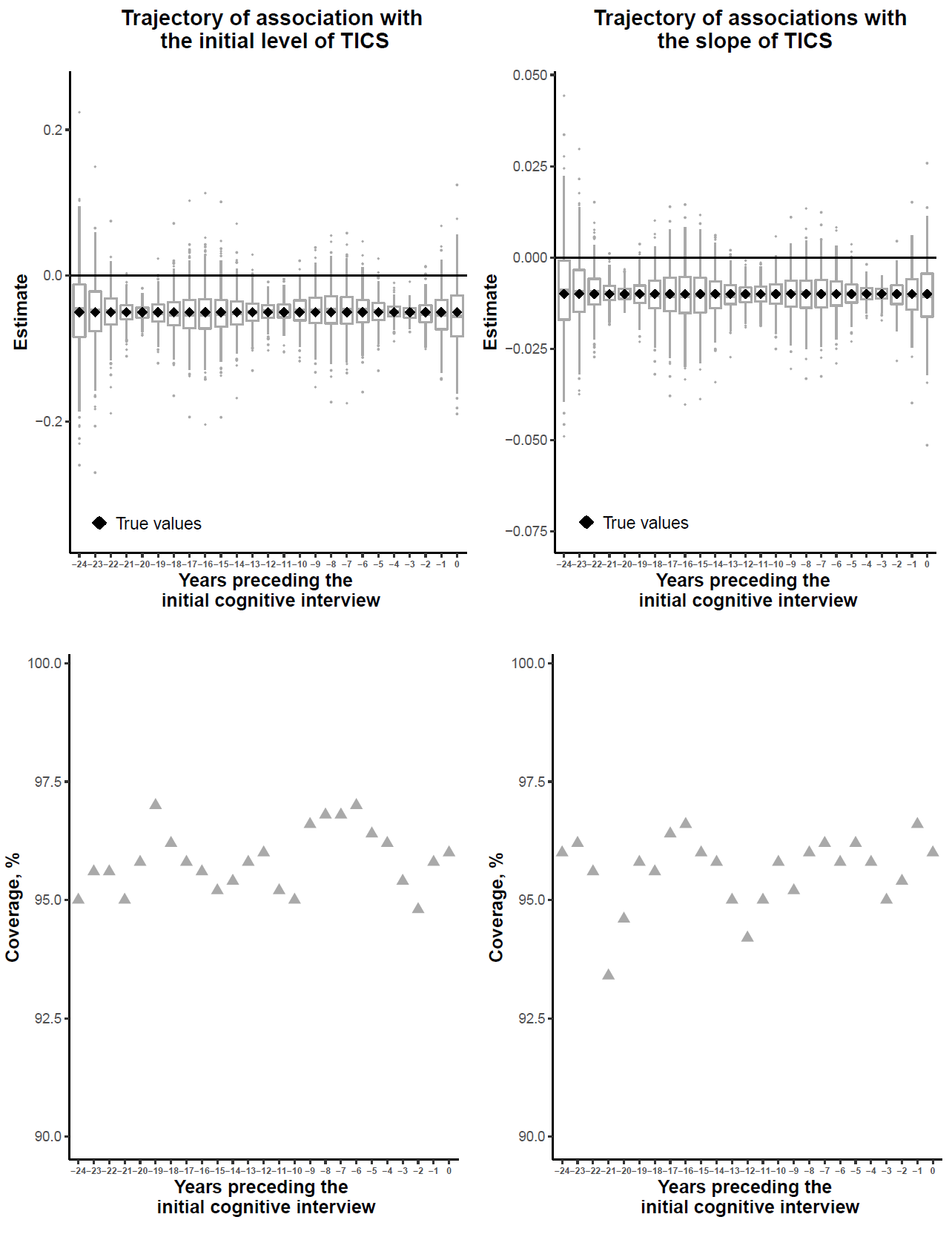

Supplement: Supplementary file 12 — Additional file 12 eFigure 10. Boxplots of the trajectory of association between the exposure history over the 24 years prior to the initial health outcome assessment on the initial level (top left panel) and slope (top right panel) of the outcome of interest when considering a larger error of measurement (i.e., σE = 1.8 instead of 0.9) across 500 simulations of 1000 subjects each, and corresponding coverage rates (low panels) for Scenario A (constant effect). [file 12874_2021_1403_MOESM12_ESM.png]

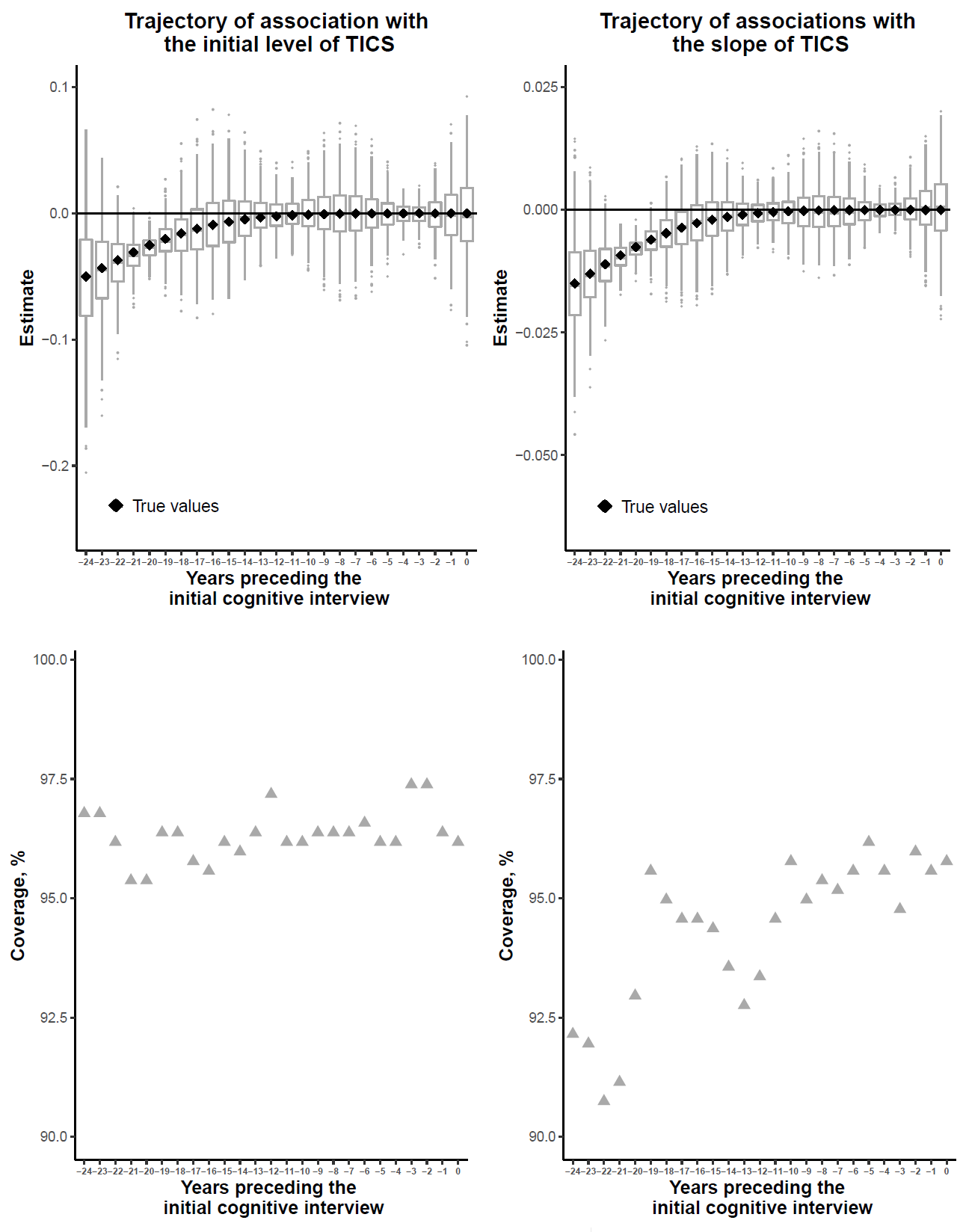

Supplement: Supplementary file 13 — Additional file 13 eFigure 11. Boxplots of the trajectory of association between the exposure history over the 24 years prior to the initial health outcome assessment on the initial level (top left panel) and slope (top right panel) of the outcome of interest when considering a larger error of measurement (i.e., σE = 1.8 instead of 0.9) across 500 simulations of 1000 subjects each, and corresponding coverage rates (low panels) for Scenario B (distant negative effect). [file 12874_2021_1403_MOESM13_ESM.png]

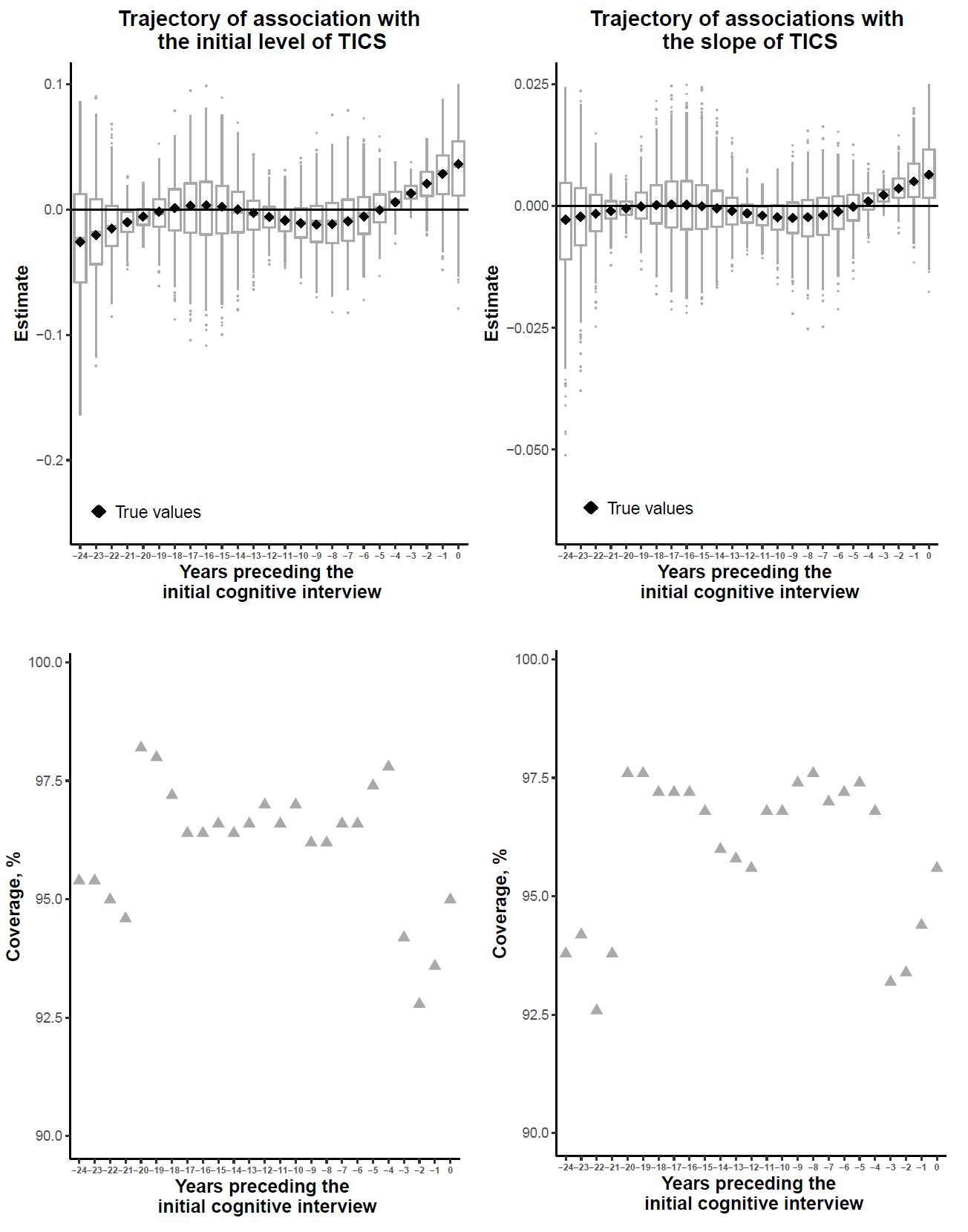

Supplement: Supplementary file 14 — Additional file 14 eFigure 12. Boxplots of the trajectory of association between the exposure history over the 24 years prior to the initial health outcome assessment on the initial level (top left panel) and slope (top right panel) of the outcome of interest when considering a larger error of measurement (i.e., σE = 1.8 instead of 0.9) across 500 simulations of 1000 subjects each, and corresponding coverage rates (low panels) for Scenario C (effect mimicking the associations between BMI and TICS in the Nurses’ Health Study). [file 12874_2021_1403_MOESM14_ESM.png]

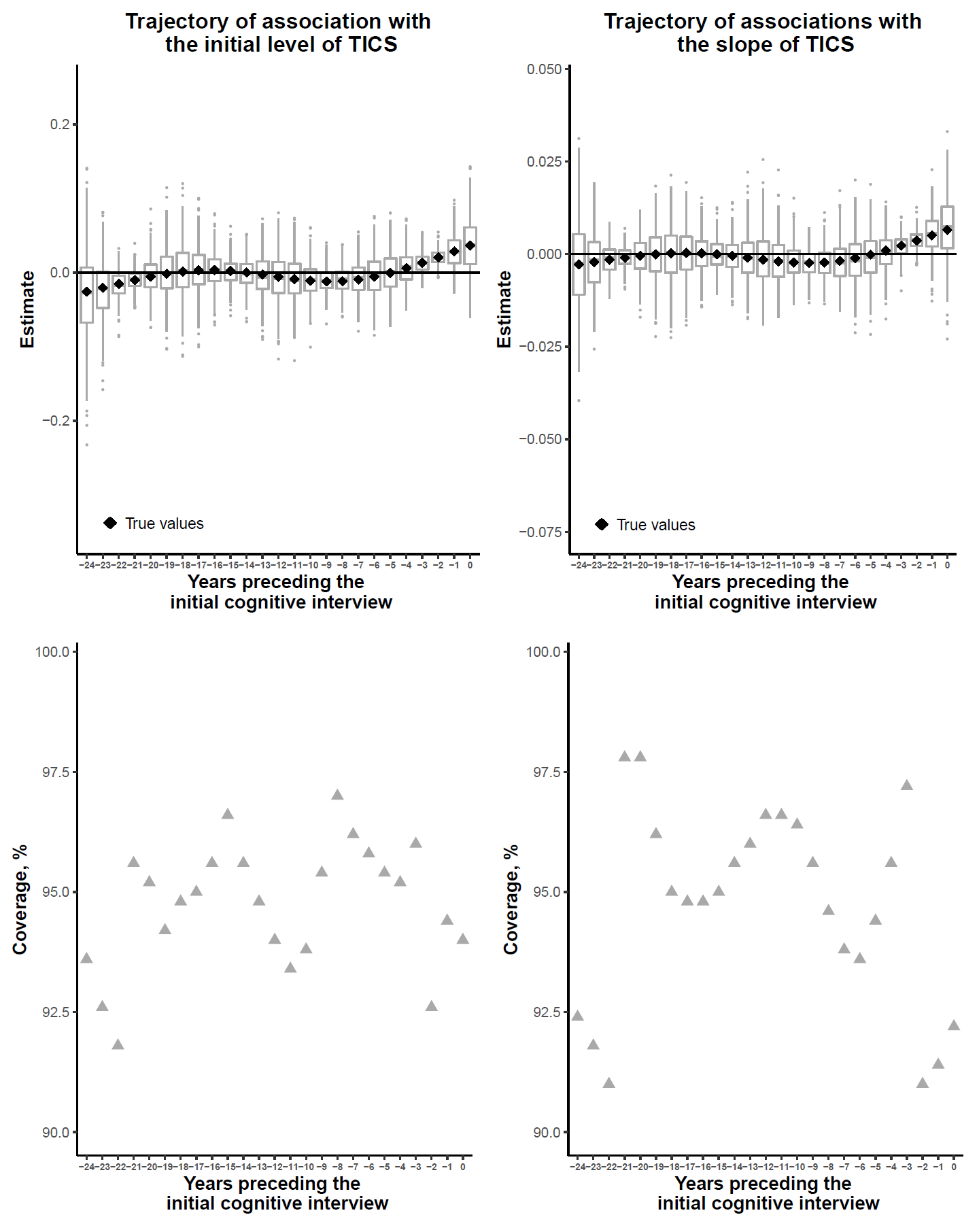

Supplement: Supplementary file 15 — Additional file 15 eFigure 13. Boxplots of the trajectory of association between the exposure history over the 24 years prior to the initial health outcome assessment on the initial level (top left panel) and slope (top right panel) of the outcome of interest when considering a higher number of inner knots of cubic splines in the definition of the BMI history (i.e., 3 inner knots located at the 25th, 50th, and 75th percentiles instead of 2 located at the 33th and 66th percentiles) across 500 simulations of 1000 subjects each, and corresponding coverage rates (low panels) for Scenario C (effect mimicking the associations between BMI and TICS in the Nurses’ Health Study). [file 12874_2021_1403_MOESM15_ESM.png]

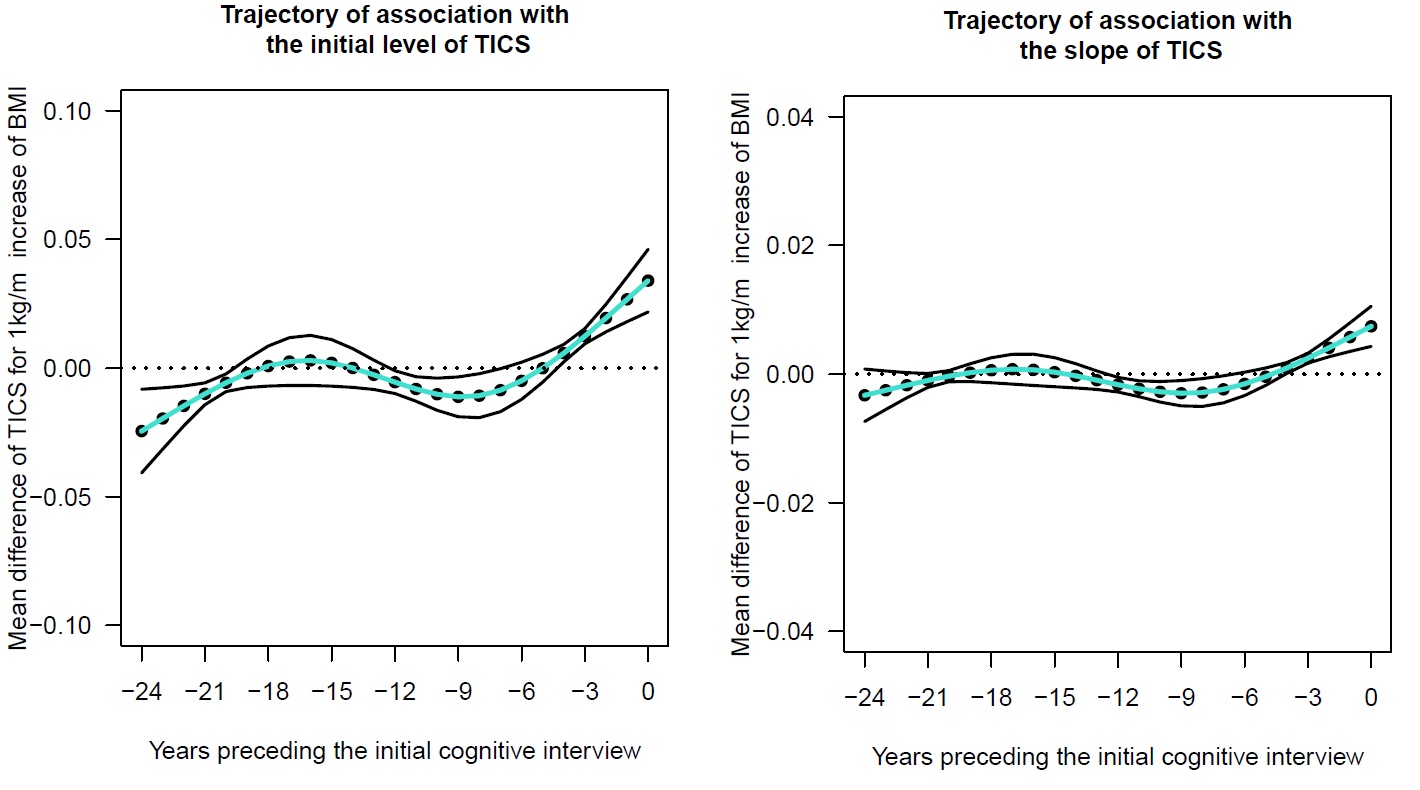

Supplement: Supplementary file 16 — Additional file 16 eFigure 14. Trajectories of associations between the body mass index history calculated every year (in black) or continuously (in blue) in the 24 years prior to the first cognitive interview on the initial level (left panel) or the slope (right panel) of the Telephone Interview for Cognitive Status (TICS) score approximated by natural cubic splines in the Nurses’ Health Study (N=19,381), United States (1976-2000). 95% confidence intervals were obtained by parametric bootstrap with 500 replicates. [file 12874_2021_1403_MOESM16_ESM.png]

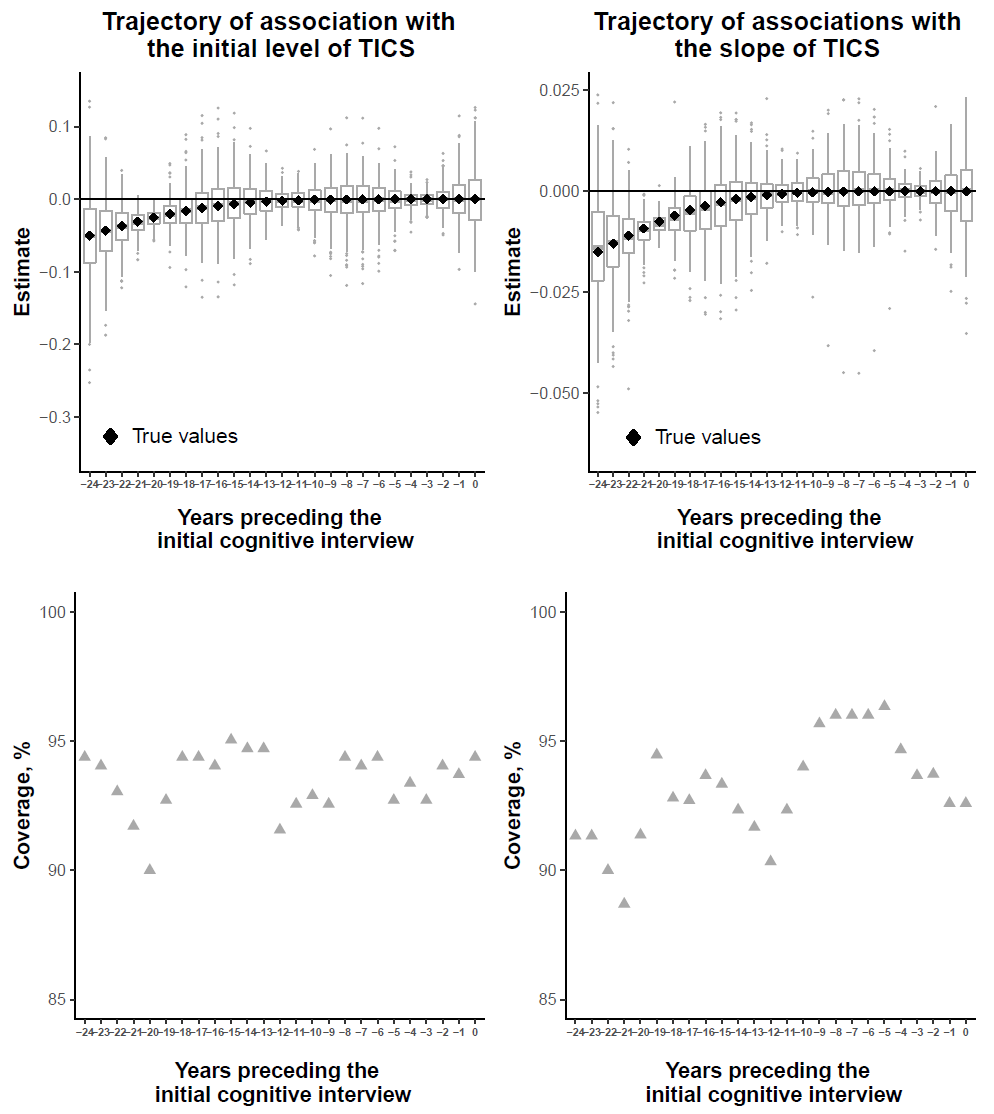

Supplement: Supplementary file 17 — Additional file 17 eFigure 15. Boxplots of the trajectory of association between the exposure history over the 24 years prior to the initial health outcome assessment on the initial level (top left panel) or the slope (top right panel) of the outcome of interest across 500 simulations of 1000 subjects each, and corresponding coverage rates of the 95% pointwise confidence interval (lower panels) for Scenario B (distant negative effect) in the context of asymmetric distribution of error measurements (Cauchy distribution with location 0 and scale 0.7). [file 12874_2021_1403_MOESM17_ESM.png]

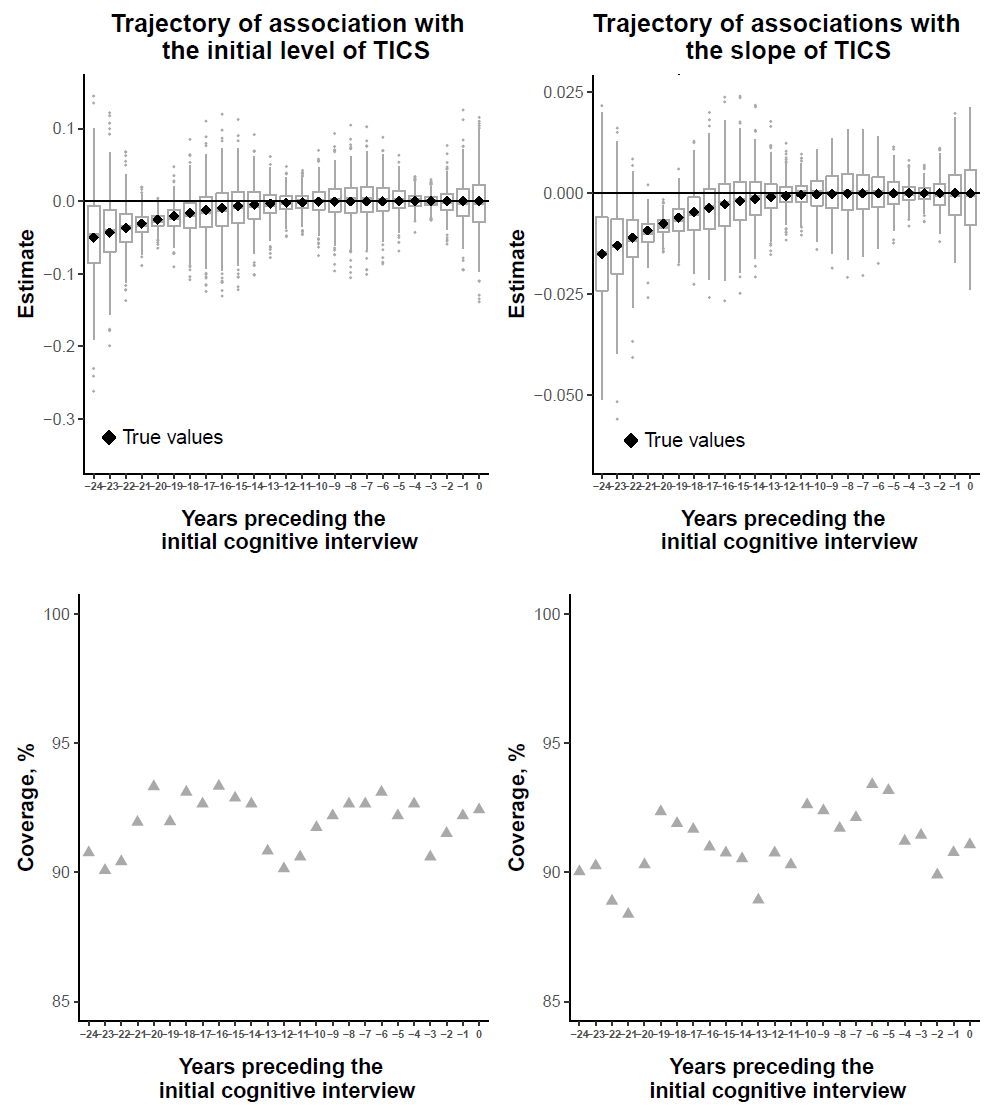

Supplement: Supplementary file 18 — Additional file 18 eFigure 16. Boxplots of the trajectory of association between the exposure history over the 24 years prior to the initial health outcome assessment on the initial level (top left panel) or the slope (top right panel) of the outcome of interest across 500 simulations of 1000 subjects each, and corresponding coverage rates of the 95% pointwise confidence interval (lower panels) for Scenario B (distant negative effect) in the context of asymmetric distribution of error measurements (Cauchy distribution with location -1 and scale 0.7). [file 12874_2021_1403_MOESM18_ESM.png]
